# Embolie pulmonaire massive hydatique

**DOI:** 10.11604/pamj.2014.19.20.5243

**Published:** 2014-09-09

**Authors:** Hicham Janah, Hicham Souhi

**Affiliations:** 1 Service de Pneumologie, Hôpital Militaire d'Instruction Mohammed V, Rabat, Maroc

**Keywords:** Embolie pulmonaire, hydatique, tomodensitométrie, pulmonary embolism, hydatid, computed tomography

## Image en médecine

Nous rapportons le cas d'un patient âgé de 39 ans d'origine rurale ayant des antécédents du kyste hydatique du foie en 1995 et d'une hydatidose pulmonaire et péricardique en 2005 traité chirurgicalement et par les antiparasitaires (A). Accusant depuis 15 jours des hémoptysies de moyenne abondance et une dyspnée d'effort. L'examen clinique trouve des râles crépitant et des cicatrices de la sternotomie et de la laparotomie. La radiographie thoracique montre une opacité hilaire droite ronde, de multiples opacités polycycliques axillaires droites et des opacités réticulaires diffuses (B). La tomodensitométrie retrouve une embolie massive par un matériel hypodense kystique intéressant la partie terminale du tronc de l'artère pulmonaire droite s'étendant sur ses branches du lobe inférieur et sur ses branches du lobe supérieur, de multiples kystes hydatiques du lobe moyen et du lobe inférieur droit et un aspect de fibrose pulmonaire bilatérale (C). L'échographie cardiaque et abdominale ne montre pas de récidive. Le bilan fonctionnel montre des troubles ventilatoires restrictifs avec une hypoxie modérée. Le patient est mis sous traitement médical antiparasitaire à base d'Albendazole 800 mg/j pendant 6 mois avec amélioration clinique et radiologique. L'embolie pulmonaire hydatique est une complication rare et grave de la maladie hydatique. Elle succède souvent à la localisation hydatique des cavités cardiaques droites ou de la VCI. Le diagnostic est facile à l'imagerie et le pronostic est très réservé.

**Figure 1:**
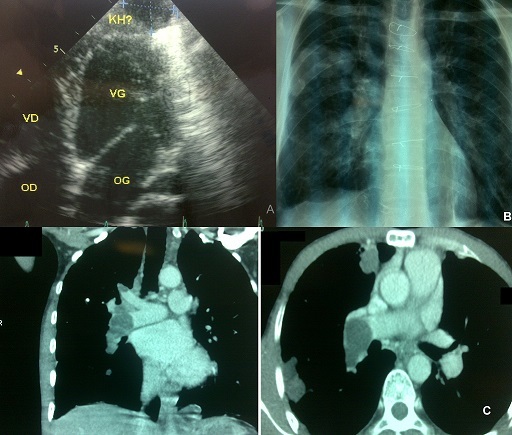
(A) échographie cardiaque montrant un kyste hydatique péricardique. (B) radiograhie thoracique montrant une opacité hilaire droite ronde, de multiples opacités polycycliques axillaires droites et des opacités réticulaires diffuses. (C) scanner thoracique montrant une embolie massive par un matériel hypodense kystique intéressant la partie terminale du tronc de l'artère pulmonaire droite s’étendant sur ses branches du lobe inférieur et sur ses branches du lobe supérieur

